# Effects of photofunctionalization on early osseointegration of titanium dental implants in the maxillary posterior region: a randomized double-blinded clinical trial

**DOI:** 10.1186/s40729-021-00318-x

**Published:** 2021-05-10

**Authors:** Bada Choi, Ye Chan Lee, Kyung Chul Oh, Jae Hoon Lee

**Affiliations:** Department of Prosthodontics, Yonsei University College of Dentistry, Seoul, 03722 Korea

**Keywords:** Photofunctionalization, Early osseointegration, Titanium dental implants, Maxillary posterior region, Bone quality

## Abstract

**Background:**

This study aimed to investigate the effects of ultraviolet (UV) photofunctionalization on the stability of implants during the early phase in the posterior region of the maxilla. The study was a randomized double-blinded clinical trial. Half of the participants received conventional commercial implants while the other half received UV-irradiated implants. The surgical sites were classified into three bone quality groups (II, III, IV) based on the grayscale value measured on cone-beam computed tomography. The values obtained from resonance frequency analysis were recorded immediately after implant placement and at 4 weeks and at 4 months postoperatively. The marginal bone level of the implants was evaluated using periapical radiographs at 4 weeks, 4 months, and 1 year postoperatively.

**Results:**

Fifty-seven implants placed in 34 participants were analyzed in this study. In group III, significant differences were observed in terms of the differences of resonance frequency analysis values at 4 weeks (*p = 0.004*) and 4 months (*p = 0.017*) postoperatively. In group II, the UV-treated group showed significantly lesser bone loss at 4 weeks post-operatively (*p = 0.037)*.

**Conclusions:**

Within the limitation of the present study, we concluded that UV surface treatment on implants may increase the initial stability in the region of the maxilla with poor bone quality.

## Background

Successful osseointegration is one of the key factors for the clinical success of dental implant treatment [1]. The rate and quality of osseointegration are intimately related to the surface characteristics of the implants: the composition, hydrophilicity, and roughness of implant surfaces play important roles in implant-tissue interaction and osseointegration [2]. Therefore, various surface treatment methods have been introduced and developed over the past years to improve the osseointegration of dental implants.

Currently, the sandblasted and acid-etched (SLA) treatment is a widely used surface treatment method for dental implants; a retrospective analysis revealed excellent 10-year survival and success rates of SLA surface implants [3]. However, the application of implant treatments remains limited in certain clinical situations [4]. Particularly, implants placed in areas where the alveolar bone is of poor quality or quantity, such as the posterior maxilla or areas that have undergone bone grafting, exhibited inferior survival and/or success rates.

Ultraviolet (UV) irradiation, or photofunctionalization, is one of the recent surface treatment methods to promote the osseointegration of implants [5]. UV treatment reportedly increases the hydrophilicity of the implant surface and decreases surface hydrocarbon by increasing the recruitment, attachment, retention, proliferation, and overall phenotype of osteogenic cells [6]. Although several clinical studies have been conducted in an attempt to elucidate the effectiveness of UV photofunctionalization, most were retrospective, and the regions of implant placement were not consistent within the oral cavity [7, 8]. Considering the inferior success rate of the implants in the posterior maxillary regions, confining the area of investigation to a specific site is necessary. Therefore, the present study aimed to investigate the effects of photofunctionalization on the stability of implants in the posterior maxillary region with poor bone quality. The null hypothesis was that no significant difference exists between the UV-treated and non-treated implants in terms of implant stability in the posterior maxillary region.

## Methods

This study involved patients who visited the Yonsei University Dental Hospital Department of Prosthodontics from March 2016 to March 2018. Before enrolment in this study, all patients were informed about all the aspects of the study and provided written informed consent. The study protocol was in accordance with the Declaration of Helsinki from 2013 and the CONSORT Statement of 2010. This research was approved by the Ethics Committee of the Yonsei University Dental Hospital (IRB No. 2-2015-0042). The detailed inclusion and exclusion criteria of the participants are summarized in Table 1.

**Table 1:** Inclusion and exclusion criteria of the study

Inclusion criteria	Exclusion criteria
Edentulous area on the posterior maxillary region	Medically compromised patients with systemic conditions that can impede implant stability and influence the treatment outcome (e.g., type II diabetes mellitus, bisphosphonate administration, immune diseases related to bone metabolism, etc.) Bone graft-requiring condition

The study was a parallel-designed randomized double-blinded clinical trial. Random allocation was performed with an equal number of participants for the experimental and control groups. The effect size was expected to be medium. Assuming a power of 0.8 and significance level of *α*=0.05, a sample size of 34 implants per group was determined. The sample size calculation was performed using GPower (Heinrich-Heine University Düsseldorf, Software-Release 3.1).

After a research assistant picked up a random number card prior to surgery, the experimental or control group was determined based on the card number according to a preformed list. The assistant received an implant fixture or fixtures (TS IV; Osstem, Seoul, Korea) which was randomly selected from each surgery case. This simple randomization procedure was conducted for each patient; therefore, the fixtures for a participant with multiple implant installations were included in the same group.

In order to predetermine the appropriate length and diameter of the implants, participants underwent a cone-beam computed tomography (CBCT) scan before the surgery. In the UV-treated group, the implant fixtures were irradiated with a UV machine (TheraBeam Affiny; Ushio Inc. Tokyo, Japan) for 15 min to achieve the desired condition as per the manufacturer’s recommendation. Subsequently, the implants were delivered to the clinician. The clinician and the patient were blinded to the fixture.

Implant placement was conducted according to conventional procedures. Immediately after the surgery, the surgical site was evaluated by panoramic and periapical radiographs. Implant stability was evaluated during the follow-up period using the resonance frequency analysis device (Osstell; Osstell AB, Göteborg, Sweden), and the implant stability quotients (ISQ) were recorded. The timepoints for the measurement were immediately after the placement of the implants, 4 weeks, and 4 months post-operatively.

Periapical radiographs of the implants were taken at each post-operative timepoint (4-week, 4-month, and 1-year post-operatively) using the paralleling technique. The digital radiographic sensor was held parallel to the long axis of the implants with an occlusal bite jig and the X-ray beam was directed perpendicular to the receptor to clearly visualize the pitches and platforms of the fixtures on the radiograph. A specialist trained in dental radiology evaluated the marginal bone level of the implants in each periapical radiograph. First, the whole length of the fixtures was measured for magnification calibration of the radiographs. Subsequently, the distance from the platform of the fixtures to marginal bone crest was recorded mesially and distally along the axis of the implants.

To exclude the statistical errors stem from the bone quality of the implant areas, subgroup distribution was performed. The surgical sites were classified into three bone quality groups (II, III, IV) based on objective evaluation of the grayscale value measured on CBCT scans and subjective evaluation by the clinician. Specifically, a grayscale value above 500 was classified as bone quality group II, between 300 and 500 as group III, and below 300 as group IV. The ISQ values were measured as per the manufacturer’s guidelines at predetermined timepoints. Subsequently, the differences between the measured values as opposed to the initial values (i.e., values obtained immediately after the surgery) were calculated for each parameter. These difference values were considered as the final ISQ difference data. Similarly, the difference between the measured marginal bone level at each timepoint was regarded as bone loss. The mean value of mesial and distal bone loss was regarded as the bone loss for each implant.

The data were compared and then analyzed within the same bone-quality subgroup using a statistical software program (IBM SPSS Statistics version 23.0; IBM Corp., Armonk, NY). The Mann-Whitney test was individually conducted to compare the ISQ level differences within each bone-quality group, with the level of significance set at *α*=0.05.

## Results

**Fig. 1:**
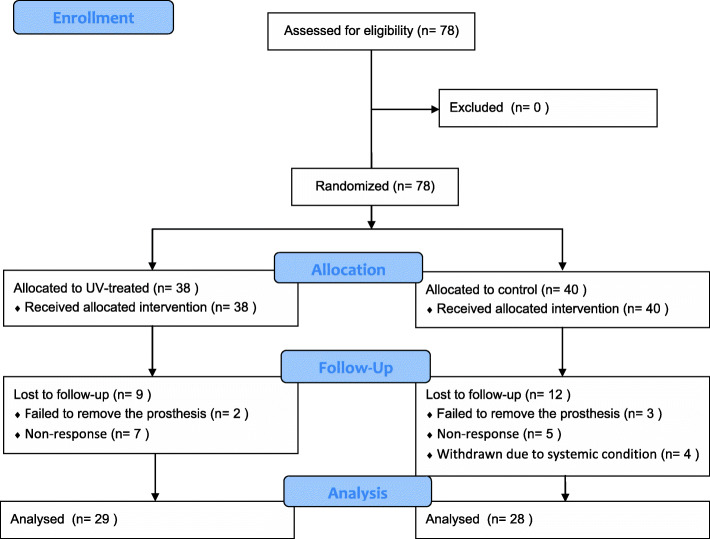
CONSORT flow chart

Seventy-eight implants were placed in 44 participants. The participants were recruited from March 2016 to March 2018 and visited the clinic for treatment and intervention at the time of randomization (baseline), and 4 weeks, 4 months, and 1 year after implantation. After the 1 year of follow-up period, 21 implants were excluded and 57 implants placed in 34 participants were analyzed in the present study (Fig. 1). The basic information of the participants in the control and experimental groups is summarized in Table 2. None of the patients had post-operative surgical complications such as dehiscence or edema. Table 3 shows subgroups composition and distribution by grayscale.

**Table 2:** Basic information of the experimental and control groups

	Experimental group (*n* = 29)	Control group (*n* = 28)
**Gender**		
Male	9	6
Female	9	10
Total	18	16
**Age, mean (range)**	64.56 (32–88 years)	67.75 (49–80 years)

**Table 3:** Subgroups composition and grayscale distribution

	Experimental group (*n* = 29)	Control group (*n* = 28)
**Bone quality (GS)**		
II (> 500)	13	7
III (300–500)	11	14
IV (< 300)	5	7
Total	29	28
**GS, mean**	466.99	442.45

**Fig. 2:**
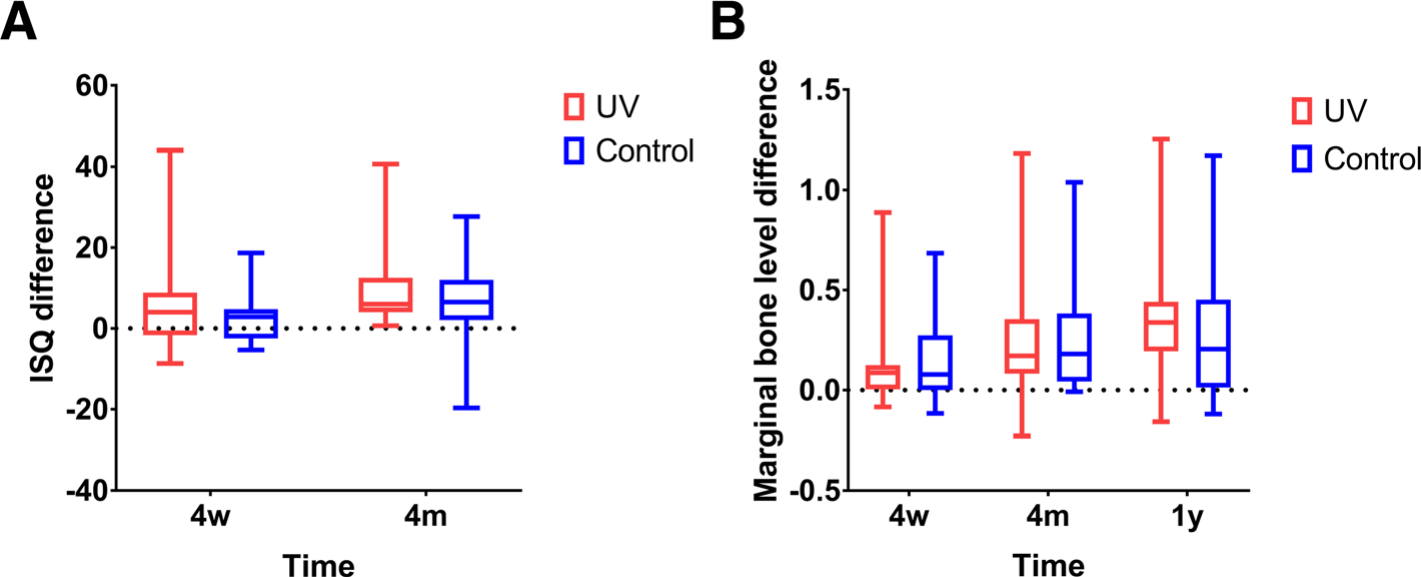
Implant stability quotient differences (**a**) and marginal bone level differences (**b**) of test versus control at each timepoint

**Fig. 3:**
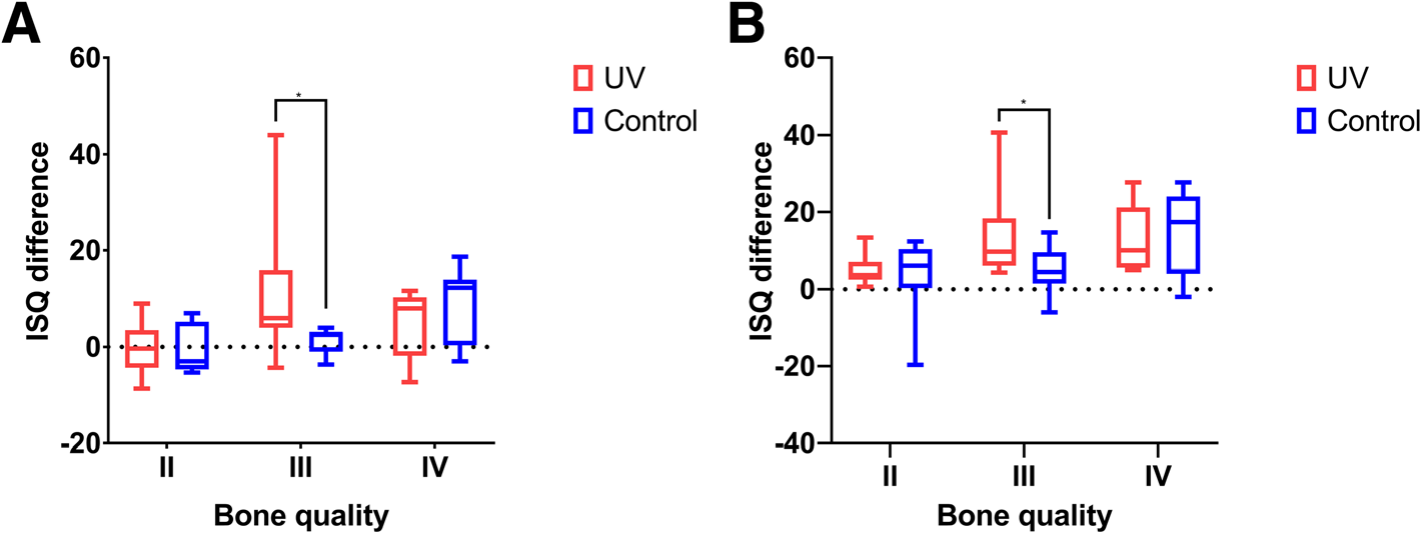
Implant stability quotient differences in each bone quality group 4 weeks (**a**) and 4 months (**b**) post-operatively

**Fig. 4:**
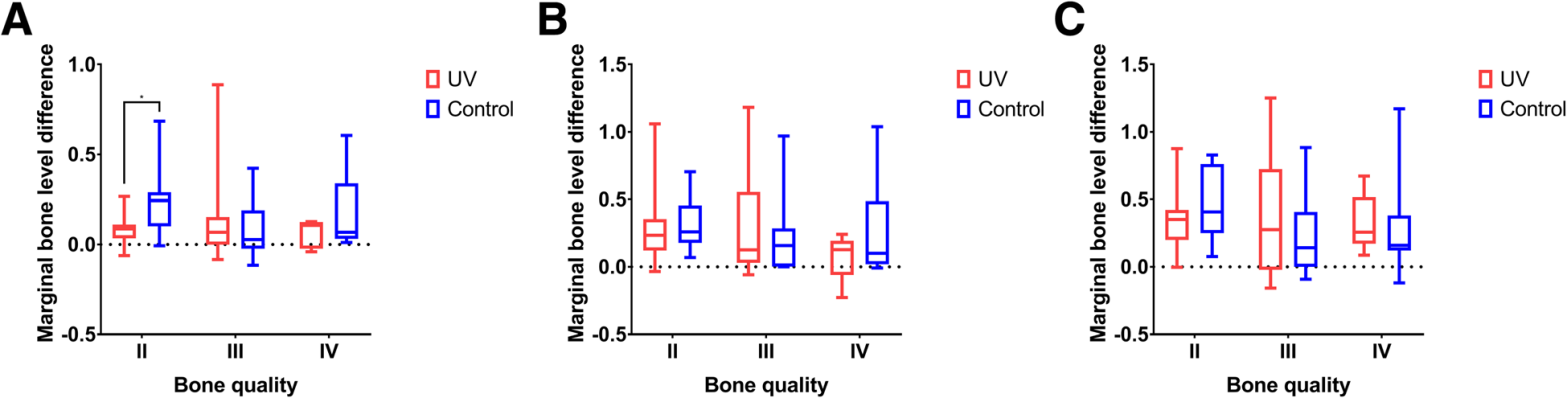
Marginal bone level differences 4 weeks (**a**), 4 months (**b**), and 1 year (**c**) post-operatively

There was no significant difference between the UV-treated and control groups in terms of ISQ differences or in marginal bone level differences (Fig. 2). In subgroup analysis, however, the ISQ differences between the UV-treated group and the control group were significantly different at 4 weeks (*p = 0.004)* and 4 months (*p = 0.017)* post-operatively in bone quality group III. The ISQ difference was significantly larger in the UV-treated group than in the control group (Fig. 3). No significant differences were observed in relation to the ISQ differences in the bone quality II and IV groups and in bone loss in other subgroups. Group II showed significantly more early bone loss (Fig. 4). The UV-treated group showed significantly lesser bone loss at 4 weeks post-operatively than the control groups. (*p = 0.037)*.

## Discussion

The null hypothesis was partially rejected as significant differences were observed between the two groups at certain timepoints. The achievement of a higher implant stability in a shorter time may enable earlier loading of dental implants, thereby facilitating the esthetic and functional outcomes. The method adopted in the present study used a simple and convenient chair-side method of photofunctionalization to rejuvenate the surface of titanium implants. As the implants were irradiated with UV light for only 15 min, the protocol did not delay the surgery time.

The bone quality should be measured and assessed to achieve a successful dental implants procedure. Based on the classification by Lekholm and Zarb, the most popular alveolar bone classification, the quality of residual alveolar bones is categorized into four types [9]. However, this classification is not digitized and depends on the surgeon’s discretion. Some authors proposed the evaluation of alveolar bone density in the presurgical planning stage using computed tomography (CT) [10, 11]. Indeed, it is possible to assess bone density using CT values (Hounsfield units: HU) and bone mineral densities obtained by CT. Although the voxel values obtained from CBCT images are not absolute, Naitoh et al. demonstrated a high-level of correlation between the voxel values of CBCT and bone mineral densities of multi-slice CT [12]. A clinical study showed that using CBCT with modified grayscale voxel values can potentially evaluate the relative bone density of the dental alveolus [13]. According to Mah et al., HU could be derived from the gray levels in dental CBCT scans using linear attenuation coefficients as an intermediate step and clinically used [14, 15]. Although some differences were observed between the actual HU and the calculated HU, the study showed that HU from CBCT images could be used as a criterion for bone quality classification in studies under unified conditions. In the present study, the grayscales of the implant areas from CBCT images were measured to classify the subgroups. After the fixtures were implanted, tetragonal lines were drawn in the preoperative CBCT images according to the diameter and length of the fixtures along the axis of the surgical sites, and the gray values in the tetragonal areas were subsequently calculated. These values, together with the surgeon’s discretion regarding the bone quality, were considered for subgroup classification.

Various evaluation tools exist for the evaluation of the osseointegration; these include push-out/pull-out test, removal torque analysis, percussion test, and histomorphometric analysis [16]. However, clinically available methods are mostly limited to resonance frequency analysis or damping capacity analysis in addition to radiographic analysis. The present study used resonance frequency analysis to record the implant stability, since the damping capacity analysis that uses Periotest (Siemens AG, Benshein, Germany) could deteriorate the stability of implants, particularly those with poor initial stability [17]. Previous studies demonstrated that the results of resonance frequency test were not correlated with the bone quality, which led to authors focussing on the differences in ISQ values at each timepoints rather than the ISQ values [18, 19].

The first intervention time for the evaluation was set 4 weeks postoperatively, as it has been reported that a “stability dip” exists at this specific timepoint [20]. The second timepoint was set at 4 months following the surgery, which is an average timepoint for implant prosthesis loading. In the present study, the UV-treated group III exhibited a remarkable increase in ISQ at the 4-week post-operative timepoint compared to the control group. These indicate that UV irradiation may dramatically increase the implant stability. Moreover, the improved ISQ value lasted until after 3 months, still presenting a significant difference compared to the control group. Hence, we can assume that once the implant stability is achieved by means of UV irradiation, it may last without complication until the prosthesis loading timepoint.

Photofunctionalization improves osteogenesis around the implant and increases interfacial bone deposition and the marginal bone seal [21]. Additionally, it induces denser cortical bone formation and a stiffer bone connection [22]. The present study showed significantly less marginal bone loss in the bone quality II group in the early stage. This result suggests that UV irradiation of the implant surface may increase the bone-to-implant contact, especially in the early stage (4 weeks), which is consistent with the findings of previous studies [21, 23]. No significant differences in the bone loss were observed between UV-treated and control implants in other subgroups or late stages. According to a previous study, after a healing period of 12 weeks, there was no difference in the bone-to-implant contact ratio between the UV-treated and control groups [24]. Marginal bone loss has a multi-factorial etiology [25]. Early crestal bone loss may not be influenced only by infection from oral microflora. In the long term, the cumulative effect of chronic etiological factors, including immunological, environmental, iatrogenic, and patient factors such as motivation, smoking, bruxism, and infection/inflammation, may influence bone loss [26]. This indicates that multiple factors can affect the 1-year results, and the effects of photofunctionalization may not be distinguishable in the late stage.

Puisys et al. conducted a clinical trial on photo-activated implants [23]. In this triple-blinded randomized controlled study, the researchers measured removal torque to assess bone-to-implant contact, suggesting that unfixed fixtures for the removal torque test fully osseointegrated after retightening. Considering the lack of clinical studies on photofunctionalization, it would be meaningful to compare their results with that of this study. With respect for the pioneers to investigate the bone-to-implant contact; however, more consensuses and ethical considerations are needed to substantiate the use of the removal torque technique in clinical trials. It seems nearly impossible to apply this removal torque method after osseointegration or to a patient plural times along the healing periods because of ethical issues. The low invasiveness and the need to continuously evaluate prognosis are the main reasons why resonance frequency- and radiographic analyses were adopted in this study. Puisys evaluated the effect of photofunctionalization in six groups, which require large sample sizes [23]. In our study, the edentulous area on the posterior maxillary region was the only inclusion criterion; therefore, the number of participants was somewhat limited. According to Puisys, photo-activated implants showed higher removal torque values at each time point than control implants, indicating better implant stability than that of control implants. This finding corresponds with the results of the present study in that remarkable enhancements were observed in ISQ values and marginal bone level at the early stages.

In this study, the null hypothesis was partially rejected as significant differences were observed in ISQ values in group III and in marginal bone level in group II at 4 weeks. The aforementioned parameters are multifactorial, and ISQ values become stable as osseointegration develops, indicating that the effect of photofunctionalization would be barely observed as time passes. Similarly, the sample size was not adequate enough to show statistically significant differences in other subgroups.

To the best of the authors’ knowledge, only a few clinical trials have evaluated the effects of photofunctionalization using a randomized control design; hence, this study is of clinical significance in this regard. However, the present study has some limitations. As the study was based on an implant-based rather than a patient-based design, potential correlation factors from the same patients could not be completely excluded. The small sample size due to restricted implant area limited the significance of out statistical results. The authors overlooked the number of the implants, whether single- or multiple-fixtures in a patient, could affect the results. One-year ISQ data were excluded because of the failure to remove the implant prosthesis or lost to follow up. For further studies, researchers should consider of applying more direct ways such as removal torque technique to assess the effect of photofunctionalization under favorable experimental condition. Also, it should be required that clinical trials aim at evaluating the implant osseointegration in sites with poorer bone quality than the posterior maxillary region, such as grafted areas, with CBCT.

## Conclusions

Based on the limitations of the present study, treatment of the implant surface with UV irradiation exhibited predictable outcomes in terms of initial stability in the posterior maxillary region. The use of UV irradiation is a simple and inexpensive method that facilitates osseointegration in compromised regions, thereby allowing a faster loading protocol.

## Data Availability

The datasets used and/or analyzed during the current study are available from the corresponding author on reasonable request.

## Abbreviations

CT: Computed tomography
CBCT: Cone-beam computed tomography
ISQ: Implant stability quotients
SLA: Sandblasted and acid-etched
UV: Ultraviolet
HU: Hounsfield unit
GS: Grayscale
